# Anal adenocarcinoma: Treatment outcomes and trends in a rare disease entity

**DOI:** 10.1002/cam4.2076

**Published:** 2019-06-07

**Authors:** Rodney E. Wegner, Richard J. White, Shaakir Hasan, Moses Raj, Dulabh Monga, Gene Finley, Alexander V. Kirichenko, James McCormick

**Affiliations:** ^1^ Division of Radiation Oncology Allegheny Health Network Cancer Institute Pennsylvania; ^2^ Division of Medical Oncology Allegheny Health Network Cancer Institute Pittsburgh Pennsylvania; ^3^ Division of Colorectal Surgery Allegheny Health Network Pittsburgh Pennsylvania

**Keywords:** anal cancer, NCDB, radiation therapy

## Abstract

**Importance:**

Primary Adenocarcinoma of the anus is a rare disease with a poor prognosis and thus tends to have a more aggressive treatment algorithm, typically involving a surgical approach. Prior to 2001, a few retrospective studies outlined improved outcomes with the incorporation of surgery with chemoradiation. However, since the publication of these studies, advancement in radiotherapy modalities and imaging have left the question of improved outcomes while reserving surgery for salvage.

**Objective:**

We conducted this National Cancer Database (NCDB)‐driven retrospective study to analyze treatment trends and outcomes in the current time from 2004 to 2015 with respect to chemoradiation and surgery.

**Design:**

Retrospective NCDB tumor registry data review—using propensity score‐adjusted multivariable analyses for survival.

**Setting:**

Database review.

**Participants:**

We selected for patients listed in the NCDB with AJCC stage 1‐3 anal adenocarcinoma diagnosed between 2004 and 2015 and selected out patients with undocumented/stage 4 disease, those with radiation outside the pelvis, not treated with systemic therapy and patients lost to follow‐up.

**Exposure(s):**

None.

**Main outcomes and measures:**

Overall survival and use of surgery in the up‐front management of anal adenocarcinoma.

**Results:**

Of the 1729 patients eligible in this study, 1028 were treated with surgery as up‐front management and 701 had definitive chemoradiation. Median overall survival for all patients was 55 months with a 5‐year survival rate of 55%. Patients treated without surgery had worse overall survival, median survival of 45 months compared to 87 months (*P *< 0.0001) with 5‐year survival rates of 42% and 55% in favor of incorporation of surgery. Analysis across patients treated with surgery alone, surgery followed by adjuvant chemoradiation, neoadjuvant chemoradiation followed by surgery, and chemoradiation alone had median survival rates of 78, 83, 92, and 46 months, respectively. Propensity score‐adjusted multivariable analysis identified older age, grade 3, high comorbidity score, and lack of surgery as predictive of worse outcome.

**Conclusions and Relevance:**

The results of the NCDB analysis indicate improved overall survival with the incorporation of surgery into the initial management of anal adenocarcinoma when compared to chemoradiation alone, despite the omission of surgery in up to 50% of the cases logged. Our results corroborate earlier studies published prior to the year 2000 for surgery to be included in the definitive management of anal adenocarcinoma.

## 1 BACKGROUND

Primary adenocarcinoma of the anal canal is a rare disease, and sometimes hard to distinguish from low‐lying rectal adenocarcinomas with local spread.^1^ Compared to the more common squamous cell carcinomas of the anal canal, adenocarcinomas have a worse prognosis.^2^ As such, the treatment algorithm for anal adenocarcinomas is more aggressive and typically involves a surgical approach, mirroring the treatment of rectal adenocarcinomas.^3^ There are more retrospective studies showing improved outcomes through the incorporation of surgery (abdominoperineal resection) either before or after chemoradiation.^4^, ^5^, ^6^ Reviewing those outcomes, one must keep in mind that many of those studies are older, and due to the rare nature of this particular disease being small and retrospective.

With that in mind, over the past 15‐20 years vast advancements have been made in imaging and radiation therapy technology, allowing higher and potentially more effective radiation doses to be delivered.^7^ With these innovations and improvements in technology, one could postulate that perhaps more attempts are being made to utilize a definitive chemoradiation approach in anal adenocarcinoma, reserving surgery for salvage. In the present study, we used the NCDB to examine trends in the up‐front treatment approach for anal adenocarcinoma, and to see if there were any differences in outcomes based on chosen treatment regimen.

## 2 METHODS AND MATERIALS

**Figure 1:**
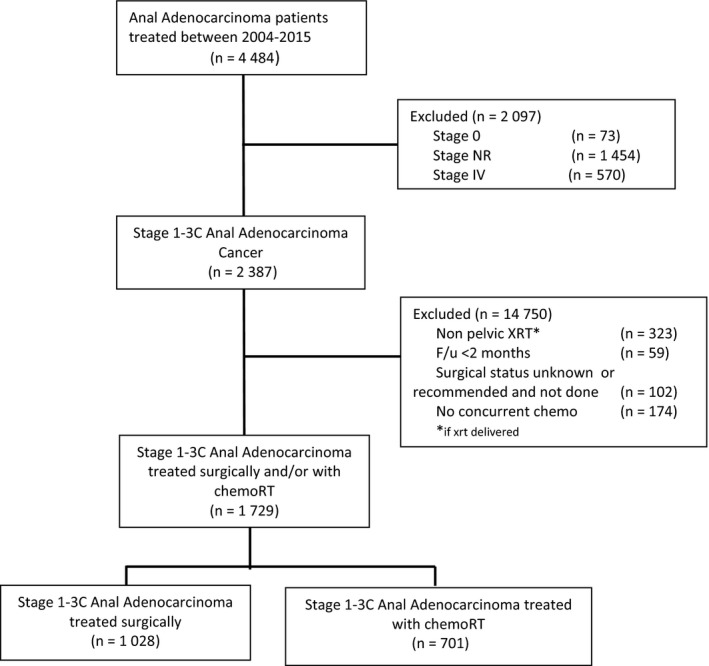
CONSORT diagram

We conducted a retrospective review using de‐identified data from the National Cancer Database (NCDB), which are exempt from IRB oversight. The NCDB is a tumor registry jointly maintained by the American Cancer Society and the American College of Surgeons for more than 1,500 hospitals accredited across the United States by the Commission on Cancer. The database is estimated to capture up to 70% of newly diagnosed malignancies each year across the country. We queried the database for patients with American Joint Committee on Cancer (AJCC) clinical stage 1‐3 anal adenocarcinoma (ICD‐0‐3 histology codes 8140 and 8480) diagnosed between 2004 and 2015. Figure 1 is a CONSORT diagram outlining the cohort selection criteria. We excluded patients with stage IV disease, undocumented stage, non‐pelvic radiation therapy, or unknown surgical status. We also excluded patients that were recommended to have surgery but refused or died prior to the operation. In addition, we excluded patients that were not treated with systemic therapy or those patients with less than 2 months of follow‐up to account for immortal time bias.

**Figure 2:**
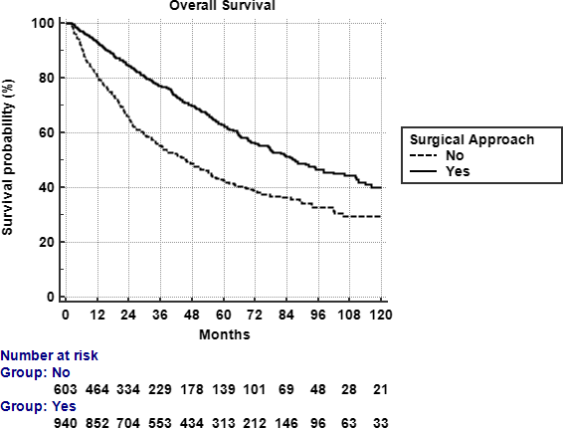
Overall Survival by surgical vs. nonsurgical approach. Five‐year survival for surgical vs nonsurgical approach was 55% and 42%, respectively (*P* < 0.0001)

Race was broken down into three broad categories: white, African American, or other. Comorbidity was quantified using the Charlson/Deyo comorbidity index.^8^ Stage was defined according to the seventh edition of the American Joint Committee on Cancer's clinical group. Socioeconomic data in the patients’ residence census tract were provided as quartiles of the percentage of persons with less than a high school education and median household income. The facility type was assigned according to the Commission on Cancer accreditation category. Locations were assigned based on data provided by the US Department of Agriculture Economic Research Service. Insurance status is documented in the NCDB as it appears on the admission page. The data used in the study are derived from a de‐identified NCDB file. The American College of Surgeons and the Commission on Cancer have not verified and are not responsible for the analytic or statistical methodology employed, or the conclusions drawn from these data by the investigator (Figure 2).

Data were analyzed using Medcalc Version 18 (Ostend, Belgium). Summary statistics are presented for discrete variables. Chi squared tests compared sociodemographic, treatment, and tumor characteristics between the treatment groups. Overall survival was calculated in months from time of diagnosis to date of last contact or death which is recorded in the NCDB. Kaplan‐Meier curves were used to calculate cumulative probability of survival.^9^ Log‐rank statistics were used to test whether there was a statistically significant difference in the cumulative proportions across groups. A Cox proportional hazards model was used for multivariable survival analysis.^10^ Due to the large nature of the dataset, factors significant on univariable analysis were entered using a stepwise backward elimination process. Adjusted hazard ratios and 95% confidence intervals are reported, using an α level of 0.05 to indicate statistical significance.

Propensity score was used to account for indication bias due to lack of randomization between patients receiving a surgical or nonsurgical approach.^11^ We defined a surgical approach as any surgery to the primary site (either up‐front followed by adjuvant therapy, after neoadjuvant chemoradiation, or alone). Multivariable logistic regression was used to calculate a propensity score indicative of conditional probability of receiving a surgical or nonsurgical approach. The propensity model included observable variables significantly associated with treatment selection on multivariable logistic regression. A Cox proportional hazards model was then constructed incorporating the propensity score, but also excluding factors included in the propensity score calculation to avoid overcorrection. The assumption of balance was further validated by stratifying the data into propensity score‐based quintiles, and confirming that the difference in propensity score mean per quintile was less than 0.10.

## 3 RESULTS

As detailed above, 1729 patients were ultimately eligible for analysis in this study; 1028 were treated with surgery as part of up‐front management and 701 were treated definitively with chemoradiation. Patient characteristics are outlined in Table 1. The vast majority of patients (77%) were stage 2 or 3 and median age was 65. In the surgical cohort, 432 (42%) had neoadjuvant chemoradiation, 179 (17%) had surgery followed by chemoradiation, and 417 (41%) had surgery alone. The median time to surgical intervention for up‐front surgery and surgery after chemoradiation was 13 days and 136 days, respectively. The median total radiation dose was 50.4 Gy over 28 fractions (interquartile range: 45‐75.6 Gy) for all patients that received radiation.

**Table 1:** Patient Demographics and Clinical Characteristics at Baseline (n = 1,729)

Characteristics	No. (%)
Sex	
Male	931 (54)
Female	798 (46)
Race	
White	1,435 (83)
African American	215 (12)
Other	79 (5)
Comorbidity Score	
0	1,373 (79)
1	265 (15)
≥2	91 (6)
Insurance	
Not Insured	74 (4)
Private Payer	625 (36)
Government	1,012 (58)
Unrecorded	18 (2)
Education %	
≥29	284 (16)
20 to 28.9	482 (28)
14 to 19.9	573 (33)
<14	377 (22)
Unrecorded	13 (1)
Treatment Facility type	
Community cancer program	197 (11)
Comprehensive community cancer program	741 (43)
Academic/research program	757 (44)
Unrecorded	34 (2)
Treatment facility location	
Metro	1,395 (80)
Urban	258 (15)
Rural	29 (2)
Unrecorded	47 (3)
Income, US dollars	
<30,000	301 (17)
30,000 to 35,000	434 (25)
35,000 to 45,999	448 (26)
>46,000	532 (31)
Unrecorded	14 (1)
Distance to treatment facility, miles	
≤10 miles	880 (51)
>10 miles	849 (49)
Age distribution, years	
≤65	918 (53)
>65	811 (47)
Year of Diagnosis	
2004‐06	300 (17)
2007‐09	425 (25)
2010‐12	474 (27)
2013‐15	530 (31)
Stage Grouping	
1	397 (23)
2	784 (45)
3	548 (32)
Grade	
Well differentiated	211 (12)
Moderately differentiated	895 (52)
Poorly differentiated	304 (18)
Not Recorded	319 (18)

The odds of receiving a surgical approach increased with younger age, white race, private insurance, lower T stage, lower N stage, and increased distance from treatment facility. A surgical‐based approach was also more likely during the years 2010‐2012. See Table 2 for complete listing of odds ratios. Figure 2 also details the percentage of patients managed with a surgical and non‐surgical approach across 2004‐2015. There was a trend toward increased surgical utilization through 2012, but that trend shifted and by 2015 more patients were being managed up‐front with definitive chemoradiation (Table 2).

**Table 2:** Comparative use of surgical vs nonsurgical approach by baseline characteristics in patients receiving treatment for anal adenocarcinoma

Characteristic	Surgical approach (n = 1,028) (%)	Chemoradiation alone (n = 701) (%)	Odds ratio	95% CI	*P*
Sex					
Male	547 (53)	384 (55)	1	Ref	
Female	481 (47)	317 (45)	1.0652	0.88‐1.29	0.52
Race					
White	871 (85)	564 (80)	1	Ref	
African American	109 (11)	106 (15)	0.67	0.50‐0.89	**0.0056**
Other	31 (4)	48 (5)	0.67	0.47‐0.96	0.99
Comorbidity Score					
0	808 (79)	565 (81)	1	Ref	
1	169 (16)	96 (14)	1.23	0.94‐1.62	0.14
≥2	51 (5)	40 (5)	0.89	0.58‐1.37	0.60
Age					
≤65	503 (49)	308 (44)	1	Ref	
>65	525 (51)	393 (56)	0.81	0.67‐0.99	**0.0412**
Insurance					
None	36 (4)	38 (5)	1	Ref	
Private payer	404 (39)	221 (32)	1.93	1.19‐3.13	**0.0078**
Government	578 (56)	434 (62)	1.41	0.87‐2.25	0.16
Unknown	10 (1)	8 (1)	1.32	0.47‐3.72	0.60
Education					
≥29%	165 (16)	119 (18)	1	Ref	
20‐28.9	287 (28)	195 (28)	1.06	0.79‐1.43	0.69
14‐19.9	342 (33)	231 (33)	1.07	0.80‐1.43	0.66
<14	229 (23)	148 (21)	1.12	0.82‐1.53	0.49
Facility type					
Community cancer program	100 (10)	97 (14)	1	Ref	
Comprehensive cancer program	440 (44)	301 (44)	1.42	1.03‐1.94	**0.03**
Academic/research program	464 (46)	293 (42)	1.54	1.12‐2.11	**0.0076**
Facility location					
Metro	830 (83)	565 (84)	1	Ref	
Urban	152 (15)	106 (15)	0.98	0.75‐1.28	0.86
Rural	19 (2)	10 (1)	1.29	0.60‐2.80	0.51
Income, USD					
<30 000	181 (18)	120 (17)	1	Ref	
30 000‐35 000	238 (23)	196 (28)	0.81	0.60‐1.08	0.15
35 000‐45 999	277 (27)	229 (25)	1.12	0.83‐1.51	0.47
>46 000	323 (32)	209 (30)	1.02	0.77‐1.37	0.87
T Stage					
1	268 (27)	99 (15)	1	Ref	
2	362 (36)	242 (36)	0.55	0.42‐0.73	**<0.0001**
3	277 (28)	229 (34)	0.45	0.33‐0.60	**<0.0001**
4	87 (9)	99 (15)	0.32	0.22‐0.47	**<0.0001**
N Stage					
0	773 (78)	457 (68)	1	Ref	
1	148 (15)	91 (13)	0.96	0.72‐1.28	0.79
2	54 (5)	80 (12)	0.39	0.28‐0.57	**<0.0001**
3	15 (2)	48 (7)	0.18	0.10‐0.33	**<0.0001**
Distance to facility					
≤10 miles	487 (47)	393 (56)	1	Ref	
>10 miles	541 (53)	308 (44)	1.42	1.17‐1.72	**0.0004**
Grade					
Well‐differentiated	137 (13)	74 (11)	1	Ref	
Moderately differentiated	571 (56)	324 (46)	0.95	0.70‐1.30	0.76
Poorly differentiated	189 (18)	115 (16)	0.89	0.62‐1.28	0.52
Not recorded	131 (13)	188 (27)	0.38	0.26‐0.54	**<0.0001**
Year of diagnosis					
2004‐06	169 (16)	131 (19)	1	Ref	
2007‐09	265 (26)	160 (23)	1.28	0.95‐1.73	0.10
2010‐12	315 (31)	159 (23)	1.54	1.14‐2.07	**0.0047**
2013‐15	279 (27)	251 (35)	0.86	0.65‐1.15	0.31

Significance of bold values is that they are *p* < 0.05.

Education is quartiles of the percentage of persons with less than a high school education in the patients’ residence census tract. Income is median household income in the patients’ residence census tract.

**Figure 3:**
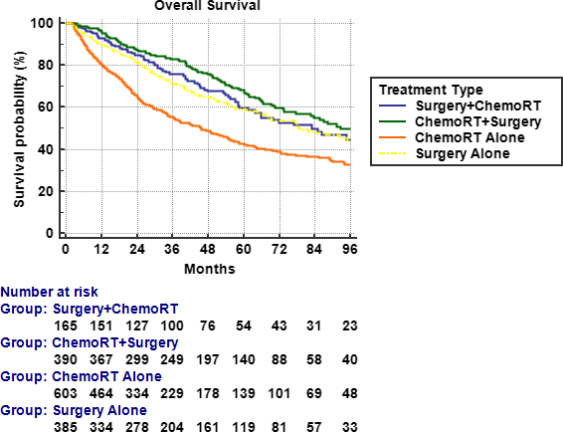
Overall Survival across treatment schema. Median survival rates for surgery + adjuvant chemoradiation, neoadjuvant chemoradiation + surgery, chemoradiation, and surgery alone were 83, 92, 45, and 69 months, respectively (*P* < 0.0001)

The median follow‐up time was 36 months (2‐152). Median overall survival for all patients was 55 months with a 5 year survival rate of 55%. On univariable analysis patients treated without surgery had worse overall survival, median survival of 87 months compared to 45 months (*P* < 0.0001). The comparative 5‐year overall survival rates were 55% and 42% in favor of incorporation of surgery (Figure 3). On multivariable cox regression, age >65, stage 3, lack of surgery, higher comorbid score, and grade 3 tumor were predictive of worse survival. Private insurance was a predictor of improved survival. See Table 3 for full results of multivariable analysis and significant predictors. As described in the methods multivariable logistic regression was used to generate a propensity score which identified chemotherapy, distance, facility type, insurance type, income, race, stage, and year of treatment as significant indicators of likelihood of surgical approach. Propensity score was generated incorporating those variables into the logistic regression. A second multivariable Cox proportional hazards model was then used including that propensity score and excluding the variables incorporated in the score generation. The propensity score‐adjusted multivariable analysis identified older age, grade 3, high comorbidity score, and lack of surgery as predictive of worse outcome (Table 4).

**Table 3:** Multivariable cox proportional hazards models for overall survival in patients receiving treatment for anal adenocarcinoma

Significant characteristic	Hazard of death (95% CI)	*P*
Cox model without propensity score		
Age		
≤65	Reference	
>65	1.96 (1.62‐2.40)	<0.0001
Comorbidity score		
0	Reference	
1	1.12 (0.91‐1.40)	0.27
≥2	1.59 (1.19‐2.13)	<0.0001
Surgical approach		
No	Reference	
Yes	0.57 (0.49‐0.66)	<0.0001
Insurance		
None	Reference	
Private	0.61 (0.49‐0.75)	<0.0001
Government	0.74 (0.49‐1.10)	0.13
Stage group		
1	Reference	
2	1.08 (0.87‐1.64)	0.48
3	1.65 (1.40‐1.93)	<0.0001
Facility type		
Community cancer program	Reference	
Comprehensive cancer program	0.95 (0.86‐1.06)	0.36
Academic/Research program	0.96 (0.73‐1.26)	0.77
Grade		
1 (well differentiated)	Reference	
2 (moderately differentiated)	1.09 (0.84‐1.44)	0.51
3 (poorly differentiated)	1.52 (1.27‐1.83)	<0.0001
Cox model with propensity score		
Age		
≤65	Reference	
>65	2.30 (1.95‐2.71)	<0.0001
Prop score	0.31 (0.16‐0.59)	0.0004
Surgical Approach		
No	Reference	
Yes	0.58 (0.50‐0.68)	<0.0001
Comorbid Score		
0	Reference	
1	1.18 (0.96‐1.46)	0.12
≥2	1.64 (1.22‐2.19)	0.0009
Grade		
1 (well‐differentiated)	Reference	
2 (moderately differentiated)	1.15 (0.88‐1.50)	0.32
3 (poorly differentiated)	1.51 (1.26‐1.81)	<0.001

CI, confidence interval.

**Table 4:** Multivariable cox proportional hazards models with propensity score for overall survival in patients receiving treatment for anal adenocarcinoma

Significant characteristic	Hazard of death (95% CI)	*P*
Cox model with propensity score		
Age		
≤65	Reference	
>65	2.30 (1.95‐2.71)	<0.0001
Prop score	0.31 (0.16‐0.59)	0.0004
Surgical approach		
No	Reference	
Yes	0.58 (0.50‐0.68)	<0.0001
Comorbid score		
0	Reference	
1	1.18 (0.96‐1.46)	0.12
≥2	1.64 (1.22‐2.19)	0.0009
Grade		
1 (well‐differentiated)	Reference	
2 (moderately differentiated)	1.15 (0.88‐1.50)	0.32
3 (poorly differentiated)	1.51 (1.26‐1.81)	<0.001

CI, confidence interval.

**Figure 4:**
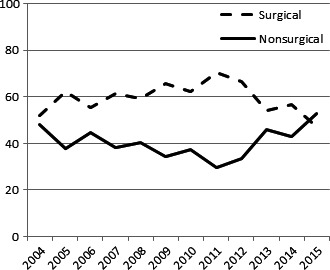
Trends in treatment approach over the years 2004‐2015

As an aside, we did do a univariable analysis across patients treated with surgery alone, surgery followed by adjuvant chemoradiation, neoadjuvant chemoradiation followed by surgery, and chemoradiation alone, showing the best outcomes with neoadjuvant chemoradiation followed by surgery (Figure 4). Median survival rates were 78, 83, 92, and 46 months, respectively (*P* < 0.0001). Furthermore, data for pathologic stage are included in the NCDB, and we did note a pathologic complete response rate of 7% in the patients treated with neoadjuvant chemoradiation followed by surgery.

## 4 DISCUSSION

Adenocarcinoma of the anal canal is quite rare compared to the more common squamous cell carcinoma, as evidenced by less than 4,500 documented cases between 2004 and 2015 in the NCDB. Compared to squamous cell carcinoma of the anal canal adenocarcinoma behaves more aggressively, and its management scheme tends to follow that of rectal adenocarcinoma as opposed to traditional anal canal.^3^ Management of squamous cell carcinoma of the anal canal has been typically treated definitively with chemoradiation going back to the favorable results seen at Wayne State University using the Nigro regimen back in the late 1970s.^12^Of interest, the original Nigro protocol only delivered a dose of 30 Gy in 15 fractions, compared to more modern regimens delivering upwards of 50 Gy.^7^, ^13^ On the other hand, rectal adenocarcinoma is now routinely treated with neoadjuvant chemoradiation to doses in the 50 Gy range, followed by surgical resection based on strong randomized data from Europe.^14^, ^15^, ^16^

Given the rarity of anal adenocarcinoma, the bulk of data to guide treatment comes from small retrospective institutional series which span many years. A small series from Memorial Sloan Kettering reviewed outcomes in 13 patients managed in various ways between 1989 and 2001 all with surgical intervention at some point in time (preop treatment followed by APR, local excision followed by adjuvant therapy, and APR followed by adjuvant treatment). Results in that cohort revealed an overall local failure rate of 37% and 2‐year overall survival of 60%.^17^ One of the largest series reviewing outcomes in anal adenocarcinoma is a multi‐institutional series from the Rare Cancer Network and included 82 patients, with an almost even split between radiotherapy and surgery (no chemotherapy) and chemoradiation alone as treatment.^4^ In this study, 5‐year overall survival was much better in the chemoradiation group compared to the radiotherapy and surgery group, 58% vs 29%, respectively; keeping in mind, chemotherapy was not delivered in the neoadjuvant group. In addition, in that study there were more older patients in the surgery arm, perhaps skewing the overall survival rates as well. In terms of local failure, rates were almost identical (~35%) between the radiotherapy/surgery group and chemoradiation group. Lastly, there have been some population‐based studies on anal adenocarcinoma, namely a SEER registry review.^5^ Despite spanning a large timeframe (1988‐2004), only 165 patients were identified with non‐metastatic anal adenocarcinoma. More than half of the patients (56%) were managed without surgery and almost 20% were treated with APR alone. At 5 years, survival rates were significantly better in the surgery groups (50%‐58%) compared to the nonoperative group (30%), a finding confirmed on multivariate analysis (*P* = 0.03).

Reviewing the literature above, it is important to keep in mind that most of those studies include patients treated in the 1980s and 1990s when imaging, staging, and radiation technology were vastly different compared to the modern era. The current standard of care for radiation treatment involving the anal canal (regardless of histology) is intensity‐modulated radiation therapy (IMRT), which allows for the delivery of a highly conformal dose of radiation to spare surrounding critical structures. Results of RTOG 0529, a phase II protocol investigating IMRT use for anal squamous cell carcinoma, confirmed significant reduction in grade 2 and grade 3 toxicity.^7^ These results have been verified by single institution data, further confirming IMRT as the current standard of care.^18^ With reduction in toxicity, it would stand to reason that patients should be able to avoid treatment interruptions and perhaps have a better nonsurgical outcome compared to those patients treated in the remote past. The improvement in technology could also explain the slight increase in a nonsurgical approach we noted in our study (>50% as of 2015) as practitioners became more comfortable with the IMRT technique. Granted, most of the above is simply hypothetical as verifying that IMRT was associated with a nonsurgical approach was not carried out in this analysis due to the likely inaccuracy of the recording of radiation technique within the NCDB.

With all of that in mind, the results of our analysis indicate that surgery should still remain a key component in the up‐front management of anal adenocarcinoma. There are, however, limitations to the data within the NCDB including its retrospective nature and inherent selection bias. In addition, the NCDB lacks important treatment details and outcomes as they relate to anal cancer, namely number of cycles of chemotherapy, type of chemotherapy, treatment‐related toxicity, and, very importantly, local failure. Salvage therapy is also not documented in the NCDB, which would be surgery for patients with local only failure from anal adenocarcinoma. We must also mention that in our series those patients with higher stage were less likely to get surgery, which would obviously skew outcome results in a negative fashion for those patients. Of note, pathologic stage is recorded in the NCDB and reviewing data after neoadjuvant chemoradiation, complete response rates were quite low, only 7%. Such a low response rate obviously lends support to the use of surgery in the management of anal adenocarcinoma, either as planned up‐front treatment or salvage.

## 5 CONCLUSIONS

The results of this NCDB analysis show improved survival with the use of surgery in the initial management of anal adenocarcinoma compared to chemoradiation alone, although surgery appears to be omitted in up to 50% of case. Despite the limitations of such reviews, our results provide further support to continue to include surgery as part of the definitive management of anal adenocarcinoma.

## CONFLICT OF INTEREST

None of the authors have any conflicts of interest to disclose.

## AUTHOR CONTRIBUTIONS

Wegner: project conception and design, data anlaysis and interpretation, drafting manuscript. Hasan: project conception and design, data analysis, manuscript revisions. White: project conception and design, manuscript revisions. Raj: Manuscript revisions, final approval. Monga: Manuscript revisions, final approval. Finley: Manuscript revisions, final approval. Kirechenko: Manuscript revisions, project conception and design, final approval. McCormick: Manuscript revisions, final approval.
